# Stromal cell ratio based on automated image analysis as a predictor for platinum-resistant recurrent ovarian cancer

**DOI:** 10.1186/s12885-019-5343-8

**Published:** 2019-02-18

**Authors:** C. Lan, J. Li, X. Huang, A. Heindl, Y. Wang, S. Yan, Y. Yuan

**Affiliations:** 1 Department of Gynecologic Oncology, Sun Yat-sen University Cancer Centre, Guangzhou, China; 2 Department of Pathology, Sun Yat-sen University Cancer Centre, Guangzhou, China; 3 State Key Laboratory of Oncology in South China, Collaborative Innovation Centre for Cancer Medicine, Guangzhou, China; 4 Centre for Evolution and Cancer, The Institute of Cancer Research, London, UK; 5 Centre for Molecular Pathology, The Royal Marsden Hospital, London, UK; 6 Division of Molecular Pathology, The Institute of Cancer Research, London, UK

**Keywords:** Automated image analysis, Stromal cell ratio, Ovarian cancer, Platinum resistant relapse

## Abstract

**Background:**

Identifying high-risk patients for platinum resistance is critical for improving clinical management of ovarian cancer. We aimed to use automated image analysis of hematoxylin & eosin (H&E) stained sections to identify the association between microenvironmental composition and platinum-resistant recurrent ovarian cancer.

**Methods:**

Ninety-one patients with ovarian cancer containing the data of automated image analysis for H&E histological sections were initially reviewed.

**Results:**

Seventy-one patients with recurrent disease were finally identified. Among 30 patients with high stromal cell ratio, 60% of the patients had platinum-resistant recurrence, which was significantly higher than the rate in patients with low stromal cell ratio (9.80%, *P* <  0.001). Multivariate logistic regression analysis revealed elevated CA125 level after 3 cycles of chemotherapy (*P* <  0.001) and high stromal cell ratio (*P* = 0.002) were the negative predictors of platinum-resistant relapse. The area under the curve (AUC) of receiver operating characteristic (ROC) curves of the models for predicting platinum-resistant recurrence with stromal cell ratio, normalization of CA125 level, and the combination of two parameters were 0.78, 0.79, and 0.89 respectively.

**Conclusions:**

Our results demonstrated stromal cell ratio based on automated image analysis may be a potential predictor for ovarian cancer patients at high risk of platinum-resistant recurrence, and it could improve the predictive value of model when combined with normalization of CA125 level after 3 cycles of chemotherapy.

## Background

Ovarian cancer is the leading cause of death in patients with gynecologic malignancies. In 2012, there were approximately 238,700 new cases of ovarian cancer and 151,900 deaths in the world [1]. Most women present with advanced disease at diagnosis. Despite initial treatment with primary debulking surgery followed by first-line taxane and platinum-based chemotherapy, about 75% of patients will eventually relapse [2]. Disease that relapses ≥6 months after completion of platinum-based chemotherapy is categorized as platinum-sensitive recurrence [3], while disease that relapses < 6 months after platinum-based chemotherapy are defined as platinum-resistant recurrence [4]. Some patients initially present with platinum-sensitive recurrent diseases, however, nearly all patients with platinum-sensitive disease ultimately develop platinum resistance, which results in death [5]. Therefore, platinum resistance is a major determinant of poor outcome in ovarian cancer. Predicting the risk of platinum resistance will provide basis of improved therapeutic efficacy in ovarian cancer.

In the past 20 years, the investigations of chemotherapy resistance were mostly restricted to cancer cell intrinsic events, and the contributory effects of different types of cells coexisting within the tumor were ignored [6]. However, a large body of evidence has shown the crosstalk between cancer cells as well as stromal cells in tumor microenvironment enable tumor growth and progression [7–11]. Moreover, microenvironment niches can promote chemoresistance within a tumor [12–15]. The abundance and characteristic of stromal cells and immune cells in tumor microenvironment have been demonstrated to lead to chemotherapy resistance in multiple cancers [7, 15–19].

Recently, computer vision techniques have been applied to tumor pathologic specimen and enable automated identification and classification of various cell types in tumor microenvironment [20–23]. These methods make rapid cell mapping in tumor pathologic specimen and quantifying cellular composition across tumor microenvironment. In one of our previous studies, we developed an automated image analysis system for identifying and quantifying heterogeneous cell types in ovarian cancer histological sections [24]. One of the findings was that high amount of stromal cells in ovarian cancer was significantly associated with poor overall survival.

Therefore, in this paper, we aimed to (i) use automated image analysis of histological sections to identify the association between microenvironmental composition and platinum-resistant recurrence in ovarian cancer, (ii) develop a novel model to predict the risk of platinum-resistant recurrence.

## Methods

### Patient selection

Ninety-one patients with ovarian cancer containing data of automated image analysis for hematoxylin & eosin (H&E) stained histological sections which were used in our previous study were initially reviewed [24]. All patients were stage III-IV disease and received primary debulking surgery in our institute.

In these 91 patients, 71 patients had recurrent disease and 20 patients did not. Only the 71 patients with recurrence were enrolled in this study. Clinical data regarding patient demographics, surgical records, pathologic characteristics, platinum-free interval, serum CA125 level (normal level defined as ≤35 U/ml), treatment regimen and follow-up were extracted from the hospital records. Patients with disease that relapses < 6 months after completion of a platinum-based chemotherapy were defined as platinum-resistant relapse. Informed consent was obtained from all patients. Ethical approval were obtained by the institutional review board of Sun Yat-sen University Cancer Center. All methods were carried out in accordance with the approved guidelines of our institute.

### Histology image analysis and microenvironmental composition quantification

Automated image analysis of H&E-stained whole-tumor sections was described in our previous study [24]. In brief, H&E histological sections of ovarian tumors were generated, digitalized and analyzed using the R package CRImage. A feature vector of 97 morphological and textural measurements derived from hematoxylin positive nuclei were all subjected to a support vector machine (SVM) for classification to identify single cells including cancer cell, stromal cell and lymphocyte. The image analysis system was trained using a small set of training data (900 cells provided by an expert pathologist) using SVM. Experiments were performed to validate the system. Then, cancer cells, stromal cells and lymphocytes were quantified in each tumor section. And the cell ratio for each sample was obtained, which was used to build predictive models in this investigation.

### Pathological scores

Pathological scores including percentage of cancer cells and stromal cells for each H&E histological section were given by a pathologist. Lymphocytic infiltration was assessed as low, median, or high infiltration.

### Statistical methods

Difference in platinum-resistant recurrence between different groups was evaluated using Chi-square test.

The receiver operating characteristic (ROC) curve was used to evaluate the performance of predictive models and was created by plotting the true positive rate (TPR) against the false positive rate (FPR) at various threshold settings. The area under the curve (AUC) provided a value description for the performance of the ROC curve. The AUC score would be between 0 and 1. The higher the value of AUC, the better the model was [25].

$$ AUC\kern0.5em =\kern0.5em \frac{\Sigma_{i\kern0.5em =\kern0.5em 1}^{n_0}\left({r}_i\kern0.5em -\kern0.5em i\right)}{n_0\times \kern0.5em {n}_1}\kern0.5em =\kern0.5em \frac{\Sigma_{i\kern0.5em =\kern0.5em 1}^{n_0}\kern0.5em {r}_i\kern0.5em -\kern0.5em \frac{n_0\kern0.5em \times \left({n}_0\kern0.5em +\kern0.5em 1\right)}{2}}{n_0\times \kern0.5em {n}_1}. $$

where *r*_*i*_ was the rank of the *i*th positive participant in the ranked list, and *n*_*0*_ and *n*_*1*_ were the numbers of the positive and negative participants.

The predictive value of models including a single parameter for platinum-resistant recurrence was determined using ROC curves. For combined parameters, the predictive models were constructed according to the following procedures. First we fitted multivariate logistic regression model using the characteristics which univariate logistic regression analysis suggested as significant and the status of platinum-resistant recurrence as the dependent variable. Then we calculated the probability *P* values of each participant using these logistic regression models. Third, we used the calculated *P* value as a predictor to build the ROC curves. The AUC between different models was compared using Z test by SAS 9.3 software. And the other data analysis were performed by SPSS Statistics 16.0 software (SPSS Inc., Chicago, IL, USA). All the test were two sided and a *p* value < 0.05 was considered as significant. The key raw data have been recorded at Sun Yat-sen University Cancer Center for future reference (number RDDA2017000306).

## Results

### Clinicopathologic characteristics for patients with recurrent ovarian cancer

The patient characteristics are presented in Table 1. In the 71 patients with recurrent ovarian cancer, 49 were platinum-sensitive relapse, and 22 were classified as platinum-resistant recurrence. Patients with elevated CA125 level after 3 cycles of chemotherapy during the front-line treatment were significantly more likely to be platinum-resistant recurrence when compared to those with normalized CA125 level after 3 cycles of chemotherapy (68.4% vs 17.3%, *P* < 0.001). There was no significant difference in age, residual disease status, FIGO stage, and histologic types between the patients with platinum-sensitive and platinum-resistant recurrence.

**Table 1:** Clinicopathologic characteristics for patients with recurrent ovarian cancer

Characteristics	Platinum-sensitive recurrence (*n* = 49) N (%)	Platinum-resistant recurrence (*n* = 22) N (%)	*P* value
Age			0.395
≤ 60 years	40 (71.4)	16 (28.6)	
> 60 years	9 (60.0)	6 (40.0)	
Residual disease			0.783
≤ 1 cm	25 (67.6)	12 (32.4)	
> 1 cm	24 (70.6)	10 (29.4)	
FIGO stage			0.115
Stage III	46 (71.9)	18 (28.1)	
Stage IV	3 (42.9)	4 (57.1)	
Histologic types			0.946
High-grade serous carcinoma	44 (69.8)	19 (30.2)	
Low-grade serous carcinoma	2 (66.7)	1 (33.3)	
Mucinous	1 (50.0)	1 (50.0)	
Endometrioid	2 (66.7)	1 (33.3)	
Normalization of CA125 level after 3 cycles of chemotherapy			< 0.001
Yes	43 (82.7)	9 (17.3)	
No	6 (31.6)	13 (68.4)	

### The association between microenvironmental composition with automated image analysis and platinum-resistant recurrence

**Fig. 1:**
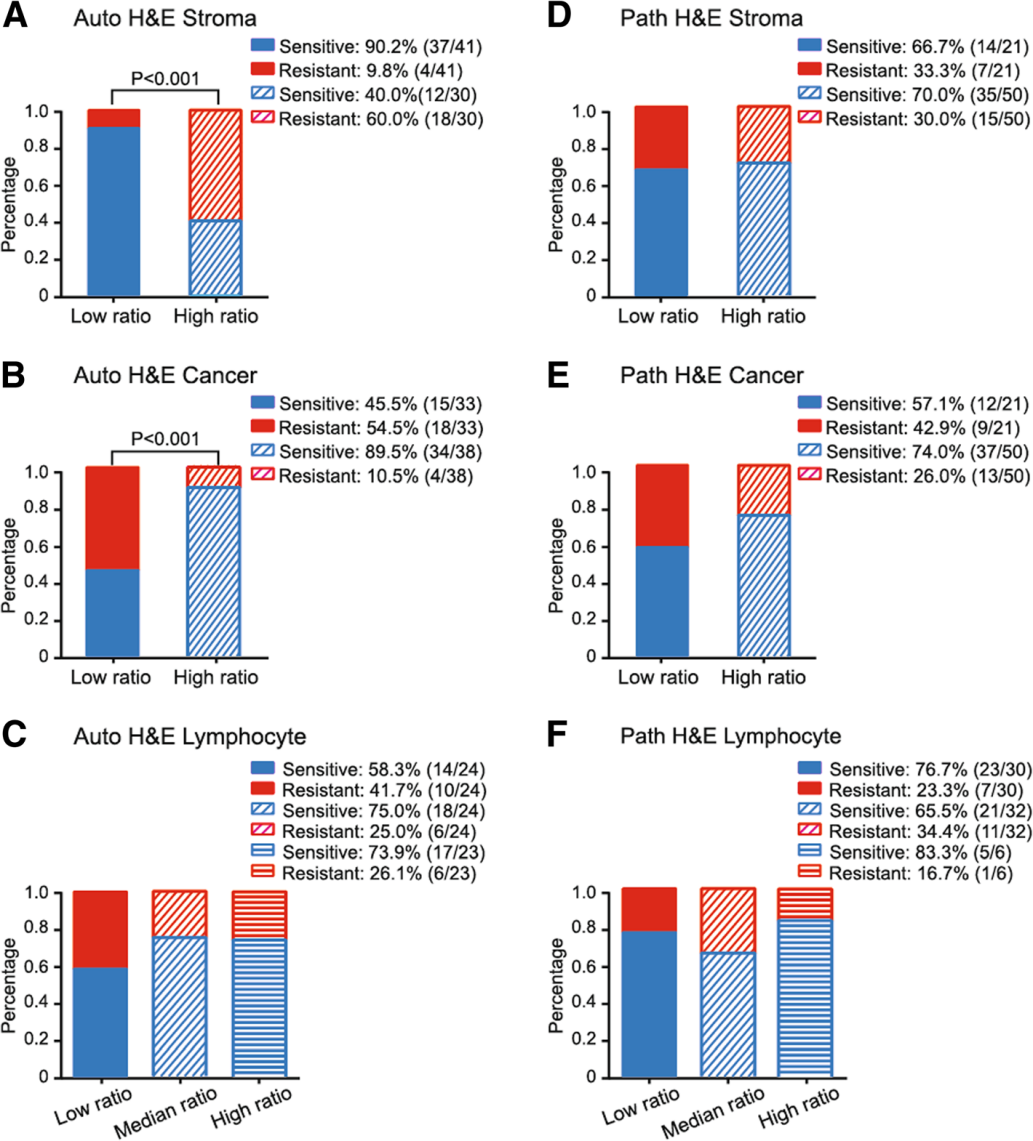
The association between microenvironmental composition (**a-c** stromal cell, tumor cell and lymphocyte ratio assessed by automated image analysis, **d-f** stromal cell, tumor cell and lymphocyte ratio assessed by pathologist’s scores) and relapse types of ovarian cancer

Microenvironmental composition was provided by automated image analysis of ovarian cancer H&E histological sections, which included the amounts of cancer cells, stromal cells and lymphocytes. Then, we tested the association between microenvironmental composition and platinum-resistant recurrence. The ROC curves were used to determine the optimal cutoff points for cell ratio to stratify patients with different recurrent type. In ROC curve analysis for the stromal cell ratio, an optimal cut-off point of 0.264 was selected (AUC = 0.78, *P* < 0.001). In high stromal cell ratio group (stromal cell ratio > 0.264), 60% (18/30) of patients were platinum-resistant recurrence, compared with only 9.80% (4/41) of patients having platinum-resistant recurrence in low stromal cell ratio group (Chi-square test *P* < 0.001) (Fig. 1a). With respect to tumor cell ratio, a best threshold of 0.683 was selected (AUC = 0.77, *P* < 0.001). The frequency of platinum-resistant recurrence in high tumor ratio group (tumor cell ratio > 0.683) was only 10.5% (4/38), which was significantly lower than the rate of platinum-resistant recurrence in low tumor ratio group (54.5%, Chi-square test *P* < 0.001) (Fig. 1b). However, ROC curve analysis could not find an optimal cut-off point regarding to lymphocyte ratio (AUC = 0.55, *P* = 0.526). Thus, we divided patients into three equal-size groups based on lymphocyte ratio according to our previous study [24]. Nevertheless, there was no significant difference in the relapse type between the low, median and high lymphocyte ratio groups (*P* = 0.379) (Fig. 1c).

### The association between microenvironmental composition assessed by pathologist’s scores and platinum-resistant recurrence

Microenvironmental composition was assessed by pathologist’s scores of H&E histological sections. The ROC curve analyses could not identify optimal thresholds for cell ratio assessed by pathologist’s scores, including cancer cell ratio (AUC = 0.53, *P* = 0.805), stromal cell ratio (AUC = 0.46, *P* = 0.647) and lymphocyte infiltration (AUC = 0.53, *P* = 0.682) to discriminate patients with different recurrent types. Therefore, we defined 25% of patients with the highest stromal cell ratio and cancer cell ratio based on pathologist’s scores as high stromal cell group and high cancer cell group, respectively. On the other hand, we divided patients into three equal-size groups based on lymphocyte infiltration. We found that stromal cell ratio and cancer cell ratio based on pathologist’s scores were not associated with the recurrent type in ovarian cancer (Fig. 1d and e). Similarly, no significant difference in recurrent types was observed between the high, median and low lymphocyte infiltration (Fig. 1f).

### Multivariate logistic regression analysis for platinum-resistant recurrent ovarian cancer

Then we determined the risk factors for platinum-resistant recurrent disease by logistic regression. Since a significantly negative correlation between cancer and stromal ratio was observed in our previous data [24], we only included the stromal cell ratio and omitted the cancer ratio in this logistic regression. The multivariate logistic regression revealed that elevated CA125 level after 3 cycles of chemotherapy (odds ratio: 18.06, *P* < 0.001) and high stromal cell ratio (odds ratio: 14.69, *P* = 0.002) were the significantly negative predictors of platinum-resistant relapse of ovarian cancer (Table 2).

**Table 2:** Logistic regression analyses on risk factors for platinum-resistant recurrent ovarian cancer

Variable	Univariate		Multivariate	
	Odds ratio (95%CI)	*P* value	Odds ratio (95%CI)	*P* value
Age				
≤ 60 years	1		1	
> 60 years	1.67 (0.51–5.45)	0.398	2.05 (0.33–12.88)	0.446
Residual disease				
≤ 1 cm	1		1	
> 1 cm	0.87 (0.32–2.38)	0.783	0.38 (0.07–2.00)	0.251
FIGO stage				
Stage III	1		1	
Stage IV	3.41 (0.69–16.76)	0.131	7.52 (0.46–121.94)	0.156
Histologic types				
High-grade serous carcinoma	1		1	
Others	1.39 (0.30–6.41)	0.673	2.05 (0.24–17.49)	0.512
Normalization of CA125 level after 3 cycles of chemotherapy				
Yes	1		1	
No	16.00 (4.66–54.91)	< 0.001	18.06 (3.69–88.38)	< 0.001
Stromal cell ratio				
Low	1		1	
High	13.88 (3.92–49.11)	< 0.001	14.69 (2.79–77.35)	0.002

### Predictive models for platinum-resistant recurrence of ovarian cancer

**Fig. 2:**
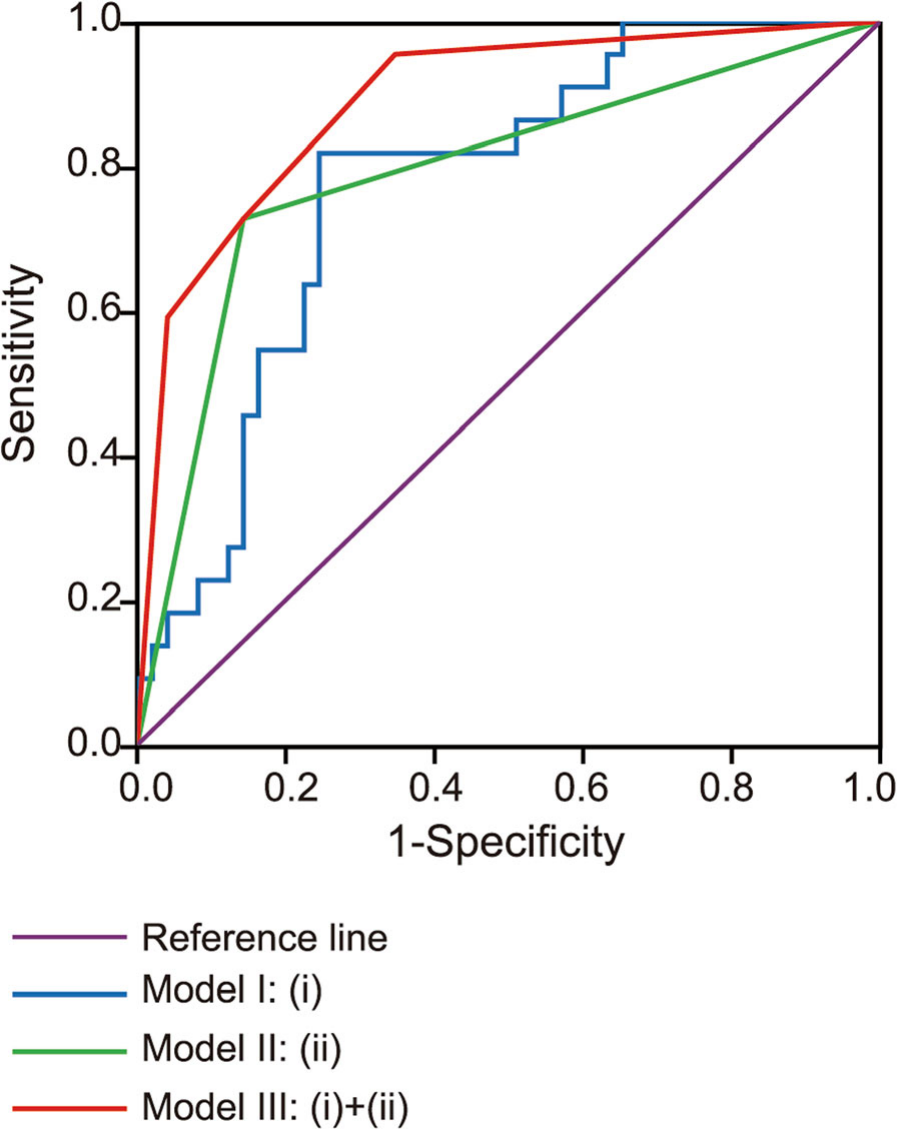
ROC curves of predictive models for platinum resistant relapse of ovarian cancer. (i) Stromal cell ratio based on automated image analysis (ii) Normalization of CA125 level after 3 cycles of chemotherapy

When the stromal cell ratio based on automated image analysis was included, the ROC curve for predicting platinum-resistant recurrence yielded a sensitivity of 81.82%, specificity of 75.51%, positive predictive value of 60.00%, and negative predictive value of 90.24%. However, the model incorporating two parameters (stromal cell ratio and normalization of CA125 level) for predicting platinum-resistant recurrence achieved a sensitivity of 95.50%, specificity of 65.30%, positive predictive value of 55.26%, and negative predictive value of 96.97% (Table 3, Fig. 2).

**Table 3:** Predictive models for platinum-resistant recurrent ovarian cancer

	Model I	Model II	Model III
Sensitivity	81.82%	72.70%	95.50%
Specificity	75.51%	85.70%	65.30%
Positive predictive value	60.00%	69.57%	55.26%
Negative predictive value	90.24%	87.50%	96.97%
AIC	70.60	68.44	56.49
Youden index	0.57	0.58	0.61
AUC	0.78	0.79	0.89
95% CI for AUC	0.66–0.89	0.67–0.92	0.81–0.97

A. Stromal cell ratio based on automated image analysis

B. Normalization of CA125 level after 3 cycles of chemotherapy

Model I: A

Model II: B

Model III: A + B

*AIC* Akaike information criterion

*AUC* Area under curve

The two-parameter model had the lowest Akaike information criterion (AIC) when compared with the model with a single parameter of stromal cell ratio and normalization of CA125 level (Table 3), which implied the combined model was the best model. The AUC-ROC of stromal cell ratio and normalization of CA125 level was 0.78 and 0.79, respectively. There was no significant difference in AUC between stromal cell ratio and normalization of CA125 level (*P* = 0.84). The AUC-ROC of the two-parameter model was 0.89. There was a marginally significant difference in AUC between the model with two parameters and with stromal cell ratio (Z = 1.61, *P* = 0.107). On the other hand, a very small trend toward significant difference in AUC was observed between the model with two parameters and with normalization of CA125 level (Z = 1.28, *P* = 0.200).

## Discussion

There is increasing evidence that heterogeneous components in tumor microenvironment collectively promote tumor chemoresistance [7, 11, 14]. However, most of studies investigated the association between microenvironmental components and chemoresistance by microarrays [26], sequencing technology [27], Polymerase Chain Reaction (PCR) [28] and immunohistochemistry [29]. Computational analysis of pathological images with quantitative and objective nature provides a new opportunity for understanding the tumor microenvironment. Several studies used automated image analysis to quantify the microenvironmental composition [20–23]. We previously applied automated image analysis on ovarian cancer and showed the stromal cell ratio was an independent prognostic factor [24]. A key advantage of this method is that it is based on fully automated image analysis of H&E-stained histology slides generated as part of clinical routine, therefore does not rely on antibodies and represents an opportunity to develop cost-effective biomarkers. However, how the quantification of the microenvironmental composition can be useful beyond prognostic biomarkers and for guiding ovarian cancer treatment remained elusive. This inspired us to design our current study, in which we proposed that a simple yet effective measure of stromal cell ratio was a potential predictive biomarker for ovarian cancer platinum treatment resistance. Here, we further demonstrated the association between microenvironmental composition and platinum-resistant relapse of ovarian cancer using automated pathological image analysis as compared to established clinical variables.

One of the important findings in this study was that high stromal cell ratio as well as low cancer cell ratio based on computational analysis of routinely generated pathological sections were significantly correlated with high frequency of platinum-resistant recurrence. The majority of stromal cells identified by image analysis were fibroblasts and myofibroblasts according to α-smooth muscle actin (α-SMA) staining which has been reported in our previous study [24]. Mhawech-Fauceglia et al. [29] revealed that the level of fibroblast activation protein alpha (FAP) expressed by fibroblasts in tumor stroma was associated with platinum resistance and shorter recurrence in ovarian cancer, which was consistent with our observation. Thus far, the mechanism underlying the acquisition of platinum resistance in stromal cells has been rarely elucidated. Carcinoma-associated fibroblasts (CAFs) are abundant and heterogeneous stromal cells in tumor microenvironment. Recently, Su et al. found that CD10^+^GPR77^+^CAFs could promote tumor formation and chemoresistance [15]. They demonstrated that mechanistically, CD10^+^GPR77^+^CAFs were driven by persistent NF-kB activation via p65 phosphorylation and acetylation, which was maintained by complement signaling via GPR77, a C5a receptor [15].

However, we could not stratify patients with distinctly different relapse types by pathologist’s scores for stromal, cancer cell ratio as well as lymphocyte infiltration. Such difference could be explained by a number of factors. Firstly, pathological scoring is highly important in research and clinical settings. However, for tasks without well-established guidelines, such as stromal cell scoring which is often not part of clinical assessment, it could be prone to variability. Secondly, because of the time-consuming nature of the task, which is to estimate the amount of stromal cells in whole-tumor section slides, the pathologist can only provide visual estimation based on scoring of five high-power fields. In contrast, automated image analysis enumerates all cells in the entire slides to provide quantitative counts, thereby relieving pathologists from repetitive tasks such as cell counting. The results suggested that automated image analysis by quantifying the amount of cells can generate accurate data and reveal tumor features correlating with treatment outcome, highlighting an opportunity for performing repetitive tasks using computers and providing a new method for studying platinum-resistant relapse of ovarian cancer.

In this study, multivariate logistic regression revealed the stromal cell ratio based on automated image analysis and normalization of CA125 level after 3 cycles of chemotherapy were the significant predictors of platinum-resistant recurrent ovarian cancer. CA125 is a widely used biomarker of ovarian cancer for evaluating disease and monitoring the treatment efficacy [30, 31]. Feng et al. found that increased preoperative CA125 level was associated with a low prognostic nutritional index (PNI), which could reflect both the nutritional and immunological status of the host [32]. Furthermore, Miao et al. reported that the PNI could help predict the platinum resistance of ovarian cancer [33]. However, limited researches investigated the direct association between the CA125 level and the platinum resistance of ovarian cancer. In the current study, the AUC-ROC of model including normalization of CA125 level after 3 cycles of chemotherapy was 0.79, with a sensitivity of 72.70% and specificity of 85.70%. It was noteworthy that the AUC-ROC was increased to 0.89 when the model containing stromal cell ratio combined with normalization of CA125 level. Although the increased AUC of two-parameter model was only marginally significant comparing with stromal cell ratio, and achieved a very small trend toward significant difference comparing with CA125 level, we considered that the number of subjects was too small to reach significant difference. Our findings implied that stromal cell ratio may be used to improve the predictive model when combined with a commonly used biomarker CA125. While this remains to be validated, early detection of high-risk patients could have significant implications for improved clinical management of ovarian cancer.

There were a number of limitations of our study, including that the lack of validation cohorts that have all these data types for this relative rare cancer type, the need for antibody staining to more closely examine diverse stromal cell populations and the need to understand the mechanisms underlying platinum resistance. Moreover, it should be noted that our data were restricted in patients with recurrent ovarian cancer, which might limit the generalizability of our results to broad population.

## Conclusion

In summary, automated image analysis quantifying microenvironmental components provides a new method for studying platinum resistant relapse of ovarian cancer. Stromal cell ratio based on image analysis may be a potential predictor for ovarian cancer patients at high risk of platinum resistant relapse. Stromal cell ratio could improve the predictive value of the model when combined with normalization of CA125 level after 3 cycles of chemotherapy.

## Abbreviations

AIC: Akaike information criterion
AUC: Area under the curve
FAP: Fibroblast activation protein
FPR: False positive rate
H&E: Hematoxylin & eosin
PCR: Polymerase Chain Reaction
ROC: Receiver operating characteristic
SVM: Support vector machine
TPR: True positive rate
α-SMA: α-smooth muscle actin
